# Characteristics of Innovators Adopting a National Personal Health Record in Portugal: Cross-Sectional Study

**DOI:** 10.2196/medinform.7887

**Published:** 2017-10-11

**Authors:** Liliana Laranjo, Inês Rodolfo, Ana Marta Pereira, Armando Brito de Sá

**Affiliations:** ^1^ Centre for Health Informatics Australian Institute of Health Innovation Macquarie University Sydney Australia; ^2^ Public Health Research Center (CISP/UNL) Portuguese School of Public Health Universidade Nova de Lisboa Lisboa Portugal; ^3^ NOVA-LINCS - Faculdade de Ciências e Tecnologia Faculty of Science and Technology Universidade Nova de Lisboa Lisboa Portugal; ^4^ Faculty of Human and Social Sciences Universidade Nova de Lisboa Lisboa Portugal; ^5^ Institute of Preventive Medicine Faculdade de Medicina Universidade de Lisboa Lisboa Portugal

**Keywords:** personal health records, diffusion of innovation, digital divide, patient participation, geographic information systems

## Abstract

**Background:**

Personal health records (PHRs) are increasingly being deployed worldwide, but their rates of adoption by patients vary widely across countries and health systems. Five main categories of adopters are usually considered when evaluating the diffusion of innovations: innovators, early adopters, early majority, late majority, and laggards.

**Objective:**

We aimed to evaluate adoption of the Portuguese PHR 3 months after its release, as well as characterize the individuals who registered and used the system during that period (the *innovators*).

**Methods:**

We conducted a cross-sectional study. *Users* and *nonusers* were defined based on their input, or not, of health-related information into the PHR. Users of the PHR were compared with nonusers regarding demographic and clinical variables. Users were further characterized according to their intensity of information input: *single input* (one single piece of health-related information recorded) and *multiple inputs*. Multivariate logistic regression was used to model the probability of being in the *multiple inputs* group. ArcGis (ESRI, Redlands, CA, USA) was used to create maps of the proportion of PHR registrations by region and district.

**Results:**

The number of registered individuals was 109,619 (66,408/109,619, 60.58% women; mean age: 44.7 years, standard deviation [SD] 18.1 years). The highest proportion of registrations was observed for those aged between 30 and 39 years (25,810/109,619, 23.55%). Furthermore, 16.88% (18,504/109,619) of registered individuals were considered *users* and 83.12% (91,115/109,619) *nonusers*. Among PHR users, 32.18% (5955/18,504) engaged in *single input* and 67.82% (12,549/18,504) in *multiple inputs*. Younger individuals and male users had higher odds of engaging in multiple inputs (odds ratio for male individuals 1.32, CI 1.19-1.48). Geographic analysis revealed higher proportions of PHR adoption in urban centers when compared with rural noncoastal districts.

**Conclusions:**

Approximately 1% of the country’s population registered during the first 3 months of the Portuguese PHR. Registered individuals were more frequently female aged between 30 and 39 years. There is evidence of a geographic gap in the adoption of the Portuguese PHR, with higher proportions of adopters in urban centers than in rural noncoastal districts.

## Introduction

Person-centered care is a pivotal element in facilitating quality improvement in health care [1]. The growing shift from paternalism to shared decision making has unveiled the importance of people’s access to their medical records and management of their health information [2,3]. Indeed, putting patients in control of their health information has been advocated as one of the solutions to the current fragmentation of health information [4,5].

Personal health records (PHRs) aim to fill the gap in personal health information management, empowering people to participate more actively in their own care. PHRs are electronic applications that enable individuals to access, manage, and share their health information in a private and secure environment [6-10]. PHRs may be classified according to their integration with the electronic health record (EHR) of a health care organization, going from *tethered* (ie, EHR-based patient portals), to stand-alone, or *untethered* PHRs (an example being Google Health, which was discontinued in 2012 for lack of widespread adoption) [2,6,9,11]. In the middle of this spectrum lie several types of hybrid PHRs, where patient-controlled functionalities are available (eg, patient-generated data entry), as well as some level of integration with EHRs [6]. Examples of hybrid PHRs include the three national government-funded PHRs that were developed in the United Kingdom, Portugal, and Australia.

Two different pathways to developing and implementing a national PHR were followed by the United Kingdom, Portugal, and Australia. In the first pathway, implemented in the United Kingdom (later discontinued) and in Portugal, the PHR is connected to a national shared record integrating EHR data from multiple providers from the National Health Service (NHS) (eg, primary health care centers and hospitals) [12-15]. In both countries, the PHRs were implemented in an opt-in model (ie, people had to actively sign up if they wanted to have an account), and the national shared records were created in an opt-out model, which means that there was implied consent for the creation of a record for each person [16]. The second pathway, followed by Australia, involves an opt-in PHR that is able to collect several summary documents from different providers without integrating them (ie, a national shared record is absent) [17].

Evidence regarding the effectiveness of PHRs for improving the quality of health care is increasing [18-21]. Published literature suggests that PHRs may lead to improvements in communication with health care providers[22-24], medication safety [24-26], medication adherence [27-30], satisfaction with care [7,22], and also in several processes of care [30-32], among other benefits. Furthermore, PHRs are increasingly being used in chronic disease management [33,34].

Nevertheless, despite the increasing deployment of PHRs by health care institutions and governments worldwide, their adoption by patients has remained slower than expected [2,12,14,35-39]. Several individual and sociotechnical factors are known to affect PHR adoption [40], such as the digital divide (ie, the gap that exists between individuals, groups, or communities in availability and use of technology) [41-43]. On the other hand, technology-related factors also play a role, such as the PHR’s design, perceived usefulness, and perceived ease of use [44,45]. Indeed, better understanding of the factors that impact PHR adoption is a crucial step in the PHR research agenda [39,46].

One theoretical framework that has been previously applied to the adoption and use of PHRs is the *diffusion of innovations* model [11]. Rogers identified five main categories of adopters with respect to their time of adoption of an innovation: innovators, early adopters, early majority, late majority, and laggards [47]. The innovators are the first group to adopt an innovation and generally correspond to 2.5% of the population in a social system [47]. Previous research has defined PHR adoption innovators as the individuals signing up for the PHR in the initial 3-month period after deployment [11]. Identifying and characterizing the innovators in PHR adoption may be an important link in delineating a strategy for the *diffusion* of this technology.

In May 2013, a Web-based national PHR was officially launched in Portugal, provided freely by the Ministry of Health. This study aimed to evaluate the adoption of the Portuguese PHR for the first 3 months after its release. A further aim was to assess registered individuals in terms of their demographic characteristics, number of health problems and medications, and frequency of PHR use to input personal health information.

## Methods

### Setting

Portugal has a National Health Service (NHS) following the principle of universal coverage. Within the NHS, access to secondary and tertiary care is mostly done through general practice referrals. The majority of primary care practices in the country use the same EHR software; the opposite is true in secondary and tertiary care. There are huge variations in terms of hardware and bandwidth speed across primary care practices [48].

Internet access and use in Portugal is lower than in several other European countries: 57% of the population has Internet access at home, and 47.1% of the population never used the Internet [49]. There is a wide gap between younger and older generations: 90.6% of individuals in the age group of 15 to 24 years use the Internet, compared with only 5% of individuals above 65 years; also, Internet use varies with educational level, with lower education being associated with lower rates of Internet use [49]. Disparities in Internet use between districts in Portugal have sharply increased between 2008 and 2014, with urban districts showing much higher rates of Internet use when compared with rural noncoastal districts [50].

Geographical disparities in Portugal are also observed in health status. There is a direct association between population health status and the coastal location and urbanization of municipalities: those with a higher score of health status are located in the western coastal line of Portugal; lowest scores are observed in rural areas [51]. Furthermore, geographical location of health care facilities unequally affects the ease of access of different groups of consumers, with poor accessibility to health services being particularly concerning for elderly individuals in less urbanized and rural areas of the country [52].

Patient involvement in health care is still relatively weak in Portugal [53], and patients’ requests to access their medical records are rare, generally involving a lengthy administrative process [54]. Less than half of the population uses the Internet for health information retrieval [55].

### Personal Health Record

The Portuguese PHR is a Web-based platform provided freely by the Ministry of Health and was officially released in May 2013 (after a 1-year period of beta-testing). At the time of data collection (end of July 2013), the PHR allowed patients to input health information (eg, health problems, chronic medication, and biometric measurements) and book primary care consultations. Integration between the PHR and the national shared record was negligible at the time of study, and very few patients had access to summary care records (SCRs), which were then being rolled out nationally (SCRs include aggregated EHR information, eg, list of health problems, current and past medications, and allergies). Furthermore, access by patients to their SCR was only possible via an e-card reader, a device rarely owned by the general public in Portugal. Planned features for future versions of the PHR included communication with health care providers, sharing data from the PHR with clinicians, prescription refills, and widespread access to SCRs.

One important factor enabling health data aggregation in Portugal is that patients registered with the NHS have a unique patient identifier (NHS number), which allows the correct integration of individual health data originating from different sources. Implementation of the PHR followed an opt-in model, which means that people had to actively register on the Web-based platform if they wanted to have an account. Access to the PHR was done through authentication with the individual NHS number and password after online registration occurred.

At the time of data collection, advertisement for the PHR was negligible, and there was no strategy in place to promote adoption. For this study, we were unable to determine the degree of public awareness regarding the existence of the PHR or to estimate the number of people reached by any communications about the PHR through mass media (eg, newspapers and radio).

### Study Design

We conducted a cross-sectional study analyzing individual-level data from registered users of the Portuguese PHR. Data were collected by the information technology (IT) services of the Ministry of Health. The dataset provided to the research team was deidentified and corresponded to the first 3 months after release (May to July 2013). The study was approved by the Ethics Committee of Lisbon Medical School.

Individual-level data from patients registered in the PHR were collected regarding age, gender, region and district of residence, chronic conditions, chronic medication, and number of times information had been entered in specific PHR fields (emergency contacts, allergies, height, weight, systolic blood pressure, diastolic blood pressure, glycemia, cholesterol, and triglycerides). Data on age, gender, and residence were automatically populated in the PHR for each patient upon registration (these administrative data are associated with each NHS number). Remaining variables were self-reported (information entered by patients in the PHR).

**Figure 1:**
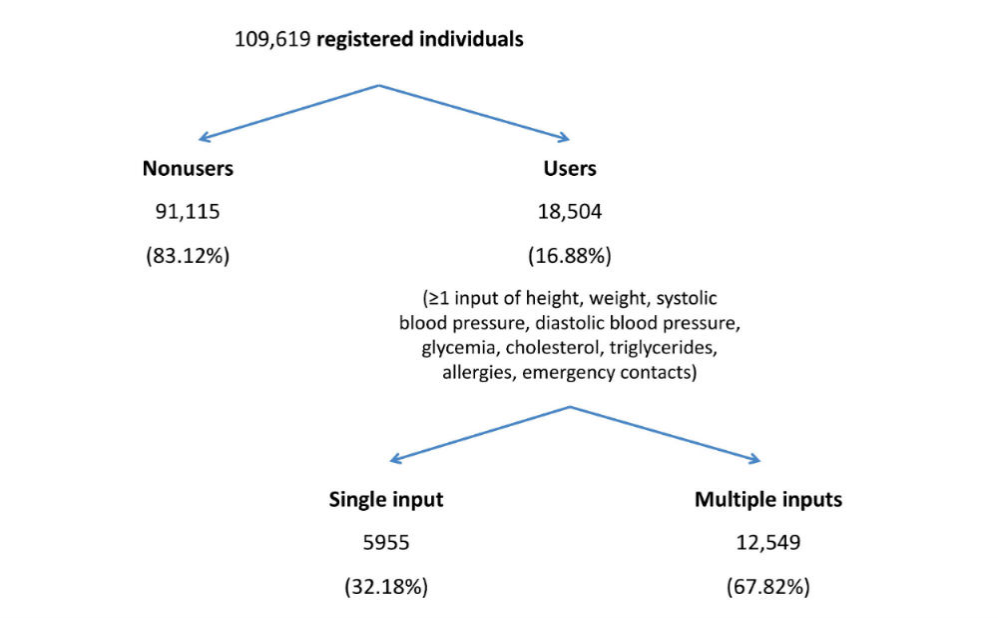
Diagram representing the classification and distribution of registered individuals into "users" and "nonusers," as well as the classification of "users" into the "single input" group and the "multiple inputs" group.

As in other studies, we used heuristic definitions to characterize subgroups of adopters according to their use of the PHR [11,56-59]. We did not use log-ins as a proxy for PHR use and focused instead on the actual input of personal health information by individuals registered in the PHR. Consequently, for the purpose of this study, a classification was created to aid the characterization of PHR adopters (Figure 1). *Registered individuals* were defined as having an account created in the PHR, independent of their actual use of the platform to input information. *Users* were defined as having entered information in at least one of the following fields: allergies, emergency contacts, height, weight, systolic blood pressure, diastolic blood pressure, glycemia, cholesterol, or triglycerides levels. *Nonusers* were defined as individuals who had signed up for an account in the PHR, but who had not entered any of those data at the time of the study. Users were further divided into *single input* and *multiple inputs*, corresponding to the recording of either one or more than one piece of information regarding any of those data fields.

### Statistical Analysis

SAS statistical software (version 9.2; SAS Institute, Inc) was used for all analyses. The distribution of continuous variables was checked for normality, and means and standard deviations (SDs) were calculated; proportions and counts were calculated for categorical variables. Univariate logistic regression models of the odds of being in the *multiple inputs* group, as a function of each individual predictor, were used to calculate crude odds ratios (ORs). Multivariate logistic regression was used to model the probability of being in the *multiple inputs* group, as a function of age category, gender, region of residence, number of health problems (categorical variable), and number of medications (categorical variable). The ArcMap functionality of ArcGis version 10 (ESRI, Redlands, CA, USA) was used to create maps of the proportion of PHR registrations by region and district.

## Results

**Figure 2:**
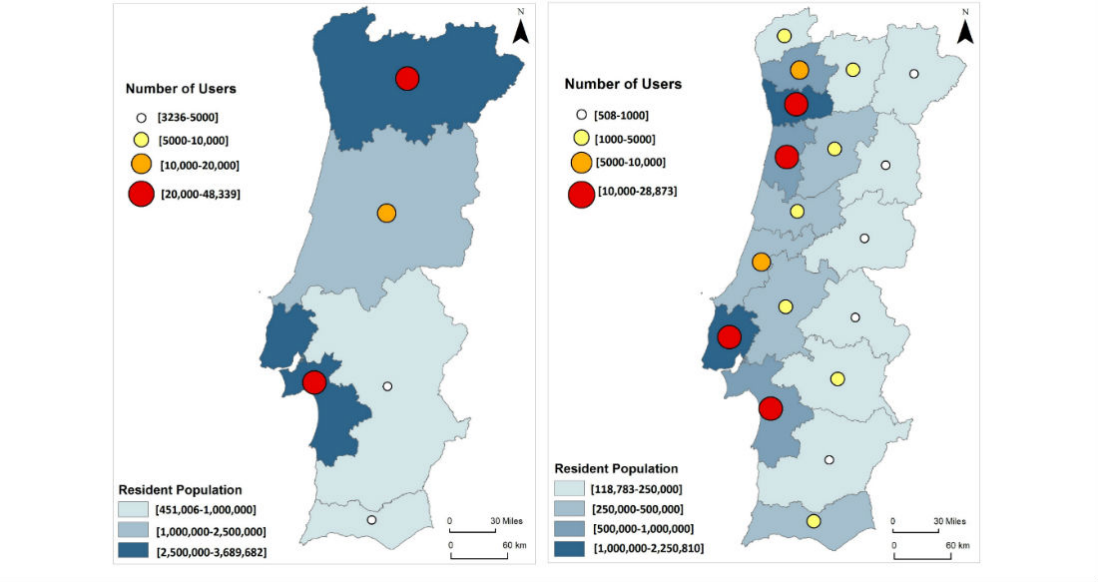
Number of patients registered in the Portuguese Personal Health Record (PHR), by region (left image) and district (right image). The right-side image (district-level data) shows higher proportions of PHR adoption (largest circles, red) in urban areas (coastal districts on the left) than in rural areas (smallest circles, white, in the noncoastal districts on the right).

We identified 109,619 individuals registered in the PHR (60.58% women; mean age: 44.7 years, SD 18.1 years), which corresponded to approximately 1% of the Portuguese population (Table 1). The highest proportion of registrations was observed in the age category from 30 to 39 years (25,810/109,619; 23.55%). Geographic analysis revealed higher proportions of PHR adoption in urban centers when compared with the rural regions of the country (ie, noncoastal areas of Portugal) (Figure 2). The districts with the highest number of registered individuals were Lisbon and Oporto (Figure 2).

Among the 109,619 registered individuals, 91,115 (83.12%) had not entered any information in the PHR regarding emergency contacts, allergies, height, weight, systolic blood pressure, diastolic blood pressure, glycemia, cholesterol, or triglycerides. This group was classified as *nonusers* (Figure 1). The remaining 18,504 individuals were classified as PHR *users*, corresponding to 16.88% of registered individuals. Users provided a total of 45,039 entries in the above-specified fields, of which, the most common were height, weight, allergies, and emergency contacts (data not shown). Users tended to be male, younger, and Lisbon residents, when compared with nonusers (Table 1).

A total of 9543 health problems and 10,913 medications were self-reported by users and nonusers (Table 1). The most commonly reported health problems were high blood pressure, diabetes, and asthma (data not shown).

PHR users were further characterized as engaging in *single input* (5955/18,504, 32.18%) or *multiple inputs* (12,549/18,504, 67.82%) (Figure 1). The differences between them, as well as the crude and adjusted ORs are illustrated in Table 2. Younger individuals had higher odds of engaging in multiple inputs, as well as male users (adjusted OR for male individuals 1.32, CI 1.19, 1.48).

**Table 1:** Characteristics of the 109,619 individuals registered in the Portuguese personal health record (PHR) according to their use of the system to input information (*nonusers* vs *users*).

Characteristic		Nonusers n (%)	Users n (%)	Total n (%)
**Age category (years)**				
	<30	18,130 (19.90)	4189 (22.63)	22,319 (20.36)
	30-40	20,157 (22.12)	5653 (30.55)	25,810 (23.55)
	40-50	17,111 (18.78)	3601 (19.46)	20,712 (18.89)
	50- 65	20,603 (22.61)	3198 (17.28)	23,801 (21.71)
	≥65	15,114 (16.59)	1863 (10.07)	16,977 (15.49)
	Total	91,115 (83.12)	18,504 (16.88)	109,619 (100.00)
**Gender**				
	Female	56,585 (62.10)	9823 (53.09)	66,408 (60.58)
	Male	34,530 (37.90)	8681 (46.91)	43,211 (39.42)
**Region**				
	Lisbon and Tagus Valley	39,925 (43.82)	8414 (45.47)	48,339 (44.10)
	North	34,486 (37.85)	6698 (36.20)	41,184 (37.57)
	Other	16,704 (18.33)	3392 (18.33)	20,096 (18.33)
	Total	91,115 (83.12)	18,504 (16.88)	109,619 (100.00)
**Health problems**				
	None	230 (21.18)	1238 (14.64)	1468 (15.38)
	1	589 (54.24)	5058 (59.81)	5647 (59.17)
	2	149 (13.72)	1017 (12.03)	1166 (12.22)
	≥3	118 (10.87)	1144 (13.53)	1262 (13.22)
	Total	1086 (11.38)	8457 (88.62)	9543 (100.00)
**Medications**				
	0	255 (15.74)	1679 (18.07)	1934 (17.72)
	1	658 (40.62)	3793 (40.82)	4451 (40.79)
	≥2	707 (43.64)	3821 (41.12)	4528 (41.49)
	Total	1620 (14.84)	9293 (85.16)	10,913 (100.00)

**Table 2:** Characteristics of users (n=18,504) according to their input with crude and adjusted odds ratios.

Characteristic^a^		Single input, n (%)	Multiple inputs, n (%)	Crude odds ratio^b^ (95% CI)	Adjusted odds ratio^c^ (95% CI)
**Age category (years)**					
	<30	1106 (18.57)	3083 (24.57)	1.46 (1.32-1.60)	1.52 (1.29-1.80)
	30-40	1694 (28.45)	3959 (31.55)	1.22 (1.12-1.33)	1.46 (1.25-1.7)
	40-50	1235 (20.74)	2366 (18.85)	(Reference)	(Reference)
	50-65	1126 (18.91)	2072 (16.51)	0.96 (0.87-1.06)	0.84 (0.71-1.0)
	≥65	794 (13.33)	1069 (8.52)	0.7 (0.63-0.79)	0.60 (0.49-0.73)
	Total	5955 (32.18)	12,549 (67.82)		
**Gender**					
	Female	3342 (56.12)	6481 (51.65)	(Reference)	(Reference)
	Male	2613 (43.88)	6068 (48.35)	1.20 (1.13-1.27)	1.32 (1.19-1.48)
**Region**					
	Lisbon and Tagus Valley	2706 (45.44)	5708 (45.49)	(Reference)	(Reference)
	North	2189 (36.76)	4509 (35.93)	0.98 (0.91-1.05)	0.95 (0.84-1.06)
	Other	1060 (17.80)	2332 (18.58)	1.04 (0.96-1.14)	1.12 (0.96-1.30)
	Total	5955 (32.18)	12,549 (67.82)		

^a^Some percentages do not total 100% due to rounding.

^b^Crude odds ratios calculated from univariate logistic regression where the probability of “multiple inputs” was modeled.

^c^Logistic regression model with predictors: age category, gender, region of residence, number of health problems, and medications.

## Discussion

### Principal Findings

To the best of our knowledge, this is the first study analyzing the adoption of a PHR in Portugal. The number of registered individuals in the Portuguese PHR 3 months after its official release was 109,619 (approximately 1% of the Portuguese population; 60.6% women; mean age: 44.7 years, SD 18.1 years), from which 16.88% (18,504/109,619) were considered *users* and 83.12% (91,115/109,619) were *nonusers*. PHR users were also characterized as engaging in *single input* (5955/18,504, 32.18%) or *multiple inputs* (12,549/18,504, 67.82%) of information related to allergies, emergency contacts, height, weight, systolic blood pressure, diastolic blood pressure, glycemia, cholesterol, or triglycerides levels. Younger individuals had higher odds of engaging in multiple inputs, as well as male users. There was evidence of a geographic gap in the adoption of the Portuguese PHR, with higher proportions of adopters in urban centers than in rural noncoastal districts.

### Comparison With Published Literature

The number of registered individuals in the Portuguese PHR 3 months after its release was 109,619, which is comparable to the adoption of other PHRs. For instance, Kaiser Permanente reported slightly higher adoption rates, with 58,734 members registering to use the site each month, on average [60]. In 2012, 4 million individuals (roughly 63% of the total number of members) had registered to use Kaiser Permanente’s PHR, making it one of the most successful and actively used PHRs in the world [61].

On the other hand, national PHRs such as the United Kingdom’s *HealthSpace* or Australia’s personally controlled EHR have not had the same success in adoption. HealthSpace, which was introduced in the English NHS in 2007, had only 172,950 registrations by the end of October 2010 and ended up being discontinued in 2012 [14]. HealthSpace allowed individuals to input health information, communicate with clinicians, and access a summary care record, but research showed that the PHR was perceived by patients as neither useful nor easy to use [14]. Similarly, Australia’s PHR had very low adoption rates since it was launched in 2012 [62]. After 3 years, with only 2 million people registered, the Australian government announced a “rescue package,” including a change of its name to “My Health Record” and the move to an opt-out model, scheduled to begin in 2018 [63,64]. As of July 2017, less than 20% (4.78 million people) of Australia’s population is registered for the My Health Record.

Our study contributes to the sparse literature on the development and implementation of national government-funded PHRs. Considering the turbulent paths of two well-known PHRs, developed in the United Kingdom and in Australia, future studies should evaluate how adoption of the Portuguese PHR is unfolding (particularly as new features become available), as well as analyze the sustainability of its use and the perceptions of patients and clinicians.

Characteristics of PHR adopters in our study are in line with findings from previous research: PHR registration and use was more frequent in women [42,65-67] and younger individuals [11], with lower registration rates and use being seen in people above 65 years [59,65,67-69]. The lower adoption by elderly patients should be further studied, as it may be associated with several different factors such as access and use of computers and the Internet, literacy, numeracy, and socioeconomic status [37]. Interestingly, studies have shown that, once enrolled, older patients were more likely to use the portal than their younger counterparts [65].

Geographic analysis revealed higher proportions of PHR adoption in urban centers than in the rural districts of the country (ie, noncoastal areas of Portugal). This divide is particularly apparent when geographical data are analyzed by district: the right-side image in Figure 2 shows that the largest circles (red), corresponding to higher proportions of PHR adoption, are located in urban areas, whereas the smallest circles (white), which indicate the lowest proportions of adoption, are located in rural areas.

The existence of an urban-rural gap raises concerns regarding the possible widening of disparities due to the digital divide [37,41,70]. In Portugal, rural areas have a higher proportion of elderly people and a less diversified network of health care services [52], showing lower scores of health status, when compared with coastal urbanized districts [51]. Additionally, Internet access and use is lower in the elderly, less educated, and those living in rural districts of the country. These groups are less likely to become adopters of a Web-based PHR, even when diffusion of this innovation spreads to early adopters and the early and late majority. Consequently, specific strategies may be needed to lessen the effects of the digital divide on existing health inequalities.

Disparities in PHR adoption have also been previously shown to be associated with race and ethnicity [42,65,67,68,70], as well as socioeconomic status, educational level [42,67,68,71], and health literacy [70], thereby raising concerns that access to this type of technology may be limited to a more socially advantaged population. For our study of PHR innovators, we were unable to access data on these types of variables. Nevertheless, if one considers the area of residence as a proxy for socioeconomic status [72], our findings reveal important disparities in PHR adoption. Given that innovators have previously been found to be similar to subsequent adopters in most sociodemographic characteristics [11], there is a concern that the urban-rural gap may be maintained as adoption of this PHR continues. Furthermore, innovators may act as opinion leaders or change agents in their communities, which could further contribute to perpetuate this gap. Research is needed to study current adoption of the Portuguese PHR, as well as to investigate its potential impact in widening health disparities.

### Implications for Clinical Practice, Health Policy, and Research

Dissemination of PHRs will facilitate change into a more patient-centered model of care. This will require a significant cultural change in Portugal, where patients’ access to their medical records and control of health information are still highly uncommon [54]. In addition, given that PHR adoption by patients is influenced by their providers’ endorsement [73], clinician involvement in the design, development, and implementation of PHRs seems crucial for their success. Despite concerns with the impact on workload, studies show that clinicians generally see several advantages in PHRs [74-76].

Continued adoption of the Portuguese PHR will depend on the availability of features that have been shown to be valued by patients, such as communication with providers, access to medical records, and administrative functionalities such as prescription refills [20,60,77-81]. For instance, registrations for Kaiser Permanente’s PHR tripled when features such as online test results and emailing a doctor’s office became available [60].

Previous studies of the Portuguese PHR found numerous usability problems, particularly in terms of readability and information architecture [80,82]. Additional studies evaluating ease of use of the current version of the Portuguese PHR are needed, as this is known to be a crucial aspect in the adoption of PHRs [11]. User-centered design strategies should help guide the development of PHR features and characteristics desired by citizens, with the aim of increasing PHR adoption [83,84].

Finally, attention should be given to the possible unanticipated consequences of the dissemination of this technology, such as the widening of inequalities and propagation of the inverse care law [85]. Therefore, ensuring universal Internet and computer access seems paramount, now that health care is increasingly reliant on IT [70]. At the same time, it is equally important to accommodate the needs of those not using or adopting this technology and make sure that they have access to the same quality of care as PHR adopters [86].

Considering the high costs associated with the development and implementation of a national PHR, independent evaluations of the implementation process should be conducted, as well an assessment of the potential value derived from this technology. Furthermore, the general public should have access to updated statistics on registrations and use of the Portuguese PHR, as well as to any evaluations that are carried out involving the system.

Future research should assess the evolution of the adoption curve, sustainability of use, perceptions of patients and clinicians, and the impact of the Portuguese PHR on process and outcome measures of health care. Furthermore, studies should evaluate the current users of the Portuguese PHR, as well as investigate possible signs of disparities between adopters and nonadopters.

### Strengths and Limitations

This study has several strengths. We studied adoption both in terms of number of registrations and actual use of the PHR to input health information, providing a comprehensive perspective on the uptake of the PHR by citizens. We were able to collect and analyze individual-level data regarding region and district of residence, which allowed for the use of geographic information systems to study the geographic distribution of PHR adoption in the country. Also, the large sample size of our study provides robustness to the results.

Some limitations should also be recognized. Data analyzed in this study correspond to the period between May and July 2013. We were only able to obtain data corresponding to 3 months after the official PHR release, which is a relatively short period of time in the adoption curve. Consequently, these results might not be generalizable to the whole population of initial adopters. Furthermore, the number of features available in the PHR at the time of release was considerably less than what is provided nowadays, a fact that should be taken into consideration when interpreting these results.

We were unable to determine the degree of public awareness regarding the existence of the PHR, which would have been important to evaluate the context in which adoption occurred.

In light of the study design, selection bias and unmeasured confounding cannot be ruled out. Potentially important variables could not be evaluated, namely socioeconomic status, access to computers, and educational level. Furthermore, collection of ethnicity data in the EHR is not permitted in Portugal, hampering a comprehensive analysis of disparities in the adoption and use of PHRs by ethnic minority groups. Data regarding health problems was available for less than 9% (n=9543) of the total sample (N=109,619). Our definitions of *users*, *nonusers*, *multiple inputs*, and *single input* were conditioned by the particularities of this specific PHR and the data that we were able to collect. We included both dynamic (eg, blood pressure) and more static (eg, allergies) types of data to define PHR use and to characterize frequency of use. The use of these different types of data in the characterization of adopters should be further studied. Data regarding the booking of primary care consultations through the PHR or the access to summary care records were not available to researchers at the time of the study. Finally, this study was limited to a specific country, so caution should be exercised when trying to generalize the results to other populations and health care systems.

### Conclusions

During the first 3 months after introducing the Portuguese PHR, 1% of the country’s population registered to use it. Registered individuals were more frequently female and aged between 30 and 39 years. There is evidence of a geographic gap in the adoption of the Portuguese PHR, with higher proportions of adopters in urban centers than in rural noncoastal districts. Future research should assess the evolution of the adoption curve and investigate possible signs of disparities between adopters and nonadopters.

## Abbreviations

EHR: electronic health record
IT: information technology
NHS: National Health Service
PHR: personal health record
SCR: summary care record
